# Effectiveness of herbal oral care products in reducing dental plaque & gingivitis – a systematic review and meta-analysis

**DOI:** 10.1186/s12906-020-2812-1

**Published:** 2020-02-11

**Authors:** Chandrashekar Janakiram, Ramanarayanan Venkitachalam, Paul Fontelo, Timothy J. Iafolla, Bruce A. Dye

**Affiliations:** 1 National Institutes of Health, National Library of Medicine and National Institute of Dental and Craniofacial Research, 31 Center Drive, Suite 4B62, Bethesda, MD 20892-2190 USA; 2 Department of Public Health Dentistry, Amrita Vishwa Vidyapeetham, Amrita School of Dentistry, Kochi, 682041 India; 3 National Library of Medicine, National Institutes of Health, 8500 Rockville Pike, Bethesda, MD 20894 USA; 4 National Institute of Dental and Craniofacial Research, 31 Center Drive, Bethesda, MD 20892-2190 USA

**Keywords:** Toothpastes, Dentifrices, Mouthwashes, Fluoride dental plaque, Herbal

## Abstract

**Background:**

Despite the large number of trials conducted using herbal oral care products for the reduction of dental plaque or gingivitis, results are conflicting and inconclusive.

**Objective:**

To assess the effectiveness of herbal oral care products compared to conventional products in reducing dental plaque and gingivitis adults.

**Methods:**

We searched the following databases for Randomised controlled trials (RCTs): MEDLINE Ovid, EMBASE Ovid etc. which yielded 493 trails. Of which 24 RCTs comparing herbal toothpaste or mouth rinse with over the counter toothpaste or mouth rinse in adults aged 18 to 65 years were included. Two authors extracted information and assessed the methodological quality of the included studies using Risk of Bias. Meta-analyses using the random-effects model were conducted for four outcomes for tooth paste and mouth rinse respectively. Mean difference (MD) or standardized mean difference (SMD) were used to estimate the effect, with 95% confidence intervals.

**Results:**

A total of 1597 adults participated in 24 RCT studies. These were classified as herbal toothpaste (HTP) (15 trials, 899 participants) and herbal mouth rinse (HMR) (9 trials, 698 participants) compared with non-herbal toothpaste (NHTP) or non-herbal mouth rinse (NHMR). We found that HTP was superior over NHTP (SMD 1.95, 95% CI (0.97–2.93)) in plaque reduction. The long-term use of NHMR was superior in reduction of dental plaque over HMR (SMD -2.61, 95% (CI 4.42–0.80)). From subgroup analysis it showed that HTP was not superior over fluoride toothpaste (SMD 0.99, 95% CI (0.14–2.13)) in reducing dental plaque. However, HTP was favoured over non-fluoride toothpaste (SMD 4.64, 95% CI (2.23–7.05)).

**Conclusion:**

For short-term reduction in dental plaque, current evidence suggests that HTP is as effective as compared to NHTP; however, evidence is from low quality studies.

## Introduction

The effective removal of dental plaque is important for maintaining periodontal and oral health [1]. Although mechanical control of microbial plaque by self-care efforts is important to prevent the plaque accumulation, this alone will not suffice. Chemical control of dental plaque is an adjunct therapy which may facilitate the removal and prevent the accumulation of microbial plaque, potentially reducing the dependence on mechanical oral care behaviours [2]. Consequently, the use of both chemical and mechanical plaque control is recommended for optimal oral hygiene [3, 4].

Various chemical agents have been used in toothpastes and mouth rinses and a few have been shown to reduce dental plaque formation [5, 6]. Due to an increased awareness of indigenous medical practices in various parts of the world, the use of “herbal” medicine has engendered interest and facilitated the growth of complementary and alternative therapies in health care promotion. Herbal ingredients have been present in oral care products, more commonly in South Asian countries, for some time [7–9]. The most common herbal ingredients to be incorporated into oral care products (e.g., toothpaste and mouth rinse) are *sanguinarine, propolis, Azadirachta indica (neem), charcoal, clove, and miswak* [10]. In the rural regions of South Asian countries, use of natural products like neem twigs, charcoal powder, and others have been an important part of regular oral hygiene practice for centuries. Many of the herbal or plant extracts have been promoted as possessing anti-inflammatory, antipyretic, analgesic, antibacterial, antiviral, anticarcinogenic and antioxidant activities by means of in vitro, in vivo, and animal studies [10, 11].

Based on these observations, several oral care product manufacturers and multinational companies have incorporated herbal ingredients into their products. Manufacturers of these products use a wide range of herbal ingredients which they claim mimic the benefits of traditional toothpastes - the ability to fight plaque, freshen breath and prevent gum disease. The tendency to “go natural” has fuelled an increase in demand for such products by consumers with many apparently opting for them because they are not tested on animals, carry no side effects, use no animal products, are vegan friendly, contains no added artificial colours or flavours, and for cultural reasons. In some regions, sale of herbal products outnumbers fluoride-based toothpastes [12] .

Comparison between herbal and conventional oral care products for the reduction of dental plaque or gingivitis were tested in clinical trials. Despite the large number of trials conducted, results are conflicting and inconclusive. Some of these products were approved by dental associations in some countries. Existing literature reviews are primarily narrative or based on single herbal ingredient (e.g., *Aloe vera*) in mouth rinse or toothpaste [13–16]. There is not a single, comprehensive systematic review which synthesized the current evidence for assessing effectiveness in reducing dental plaque and gingivitis using herbal oral care products such as herbal toothpaste (HTP) and herbal mouth rinse (HMR). Therefore, the objective of this study is to systematically assess the literature and quantitatively measure the effectiveness of herbal toothpaste and mouth rinse compared to conventional over the counter (OTC) products in reducing dental plaque and gingival inflammation in adults.

## Materials and methods

This systematic review was conducted following the preferred reporting items for systematic reviews and meta-analyses (PRISMA) statement and the patient, intervention, comparison, outcomes (PICO) method as applicable in relation to the topic of the review:

*Patient*: adults > 18 years.

*Intervention:* Herbal Toothpastes or Mouth rinses.

*Comparison*: Over the counter (OTC) non-herbal oral care products (Fluoride toothpaste, Non-fluoride/Non-herbal toothpaste, Chlorhexidine mouth rinse or non-herbal Mouth rinse).

*Outcomes*: Reduction in dental plaque levels and gingival inflammation.

*Focused question*: Are the herbal care products (toothpaste and mouth rinse) non-inferior in reduction of dental plaque and gingival inflammation over the commercial over the counter (OTC) products in adults?

### Eligibility criteria

This systematic review was limited to randomized controlled trials with parallel arm design (RCTs) where randomization occurred at the level of the individual. Quasi-randomized trials were excluded. Included studies were those with participants who were adults > 18 years with no other restrictions on age or gender nor study conduct in any country. The intervention group consisted of subjects using herbal oral care products (either toothpaste or mouth rinse) which had an active herbal ingredient, or a natural or plant extract as claimed by the manufacturer. The control group consisted of subjects (active controls) using formulation containing non-herbal active ingredients in toothpaste and mouth rinse that were commercially available OTC or manufactured as placebos for the study.

### Outcomes

The following outcomes were assessed for both the intervention arm (HMR and/or HTP) and the control arm of the studies:

1. Mean reduction in the plaque measure by Silness and Loe Plaque index or modified Quigley Hein plaque index;
2. Mean reduction of the gingival inflammation by Loe and Silness Gingival index;
3. Short-term effects (studies with 4-week follow-up acceptability range ± 3 days)
4. Long-term effects (studies with 12-weeks follow-up acceptability range ± 3 days)

### Information sources and search

The electronic search was performed with the databases MEDLINE Ovid, EMBASE Ovid, WHO clinical trial register, ClinicalTrials.gov and Cochrane Library, with a platform-specific search strategy consisting of combinations of controlled terms (MeSH) and text words. A copy of the detailed search strategy for MEDLINE Ovid is included in Additional file 1: Table S1. Additionally, the bibliographies of retrieved articles were reviewed. The search strategy terms included “herbal mouth rinses” or herbal tooth pastes” with no language restrictions. Two authors (CJ and RV) independently eliminated any duplicate from the gathered results and examined the remaining articles by title and abstract. Subsequently, the full texts were obtained and analysed for further inclusion/exclusion. Studies that did not meet the inclusion criteria were excluded. The article full-text of those identified after the title and abstract were screened. The search was performed on June 2018 for all mentioned databases. There was no lower limit for the analysed time frame.

### Data collection process and data items

For every included study, using Microsoft Excel sheet, the participant study definition, risk of bias assessment, total length of the study, unit of randomization, unit of analysis, participants’ characteristics, interventions, outcomes, results and other items were collected for each study by two reviewers. The treatment effect for each study was summarized using mean differences and standard deviations (SD). The standardized weighted-mean differences (SMD) were calculated for outcomes (measured by different scales/indices) for each study. Random-effects models [17] were used to calculate a pooled estimate of effect and its 95% confidence intervals (CIs). Authors were contacted in the event of missing data. Non-reported SDs were calculated from the reported standard errors, confidence intervals, presented for mean differences. Data were analysed with RevMan 5.3.

### Assessment of risk of bias

The risk of bias assessment of the included studies used the approach recommended by with the Cochrane Collaboration’s tool [18]. All included studies were assessed independently and in duplicate by two review authors (CJ and RV) for study design characteristics and features of internal validity. Assessment was done within and across studies. The first step was writing a description of the results of each included study. Next, involved was the assessment of the risk of bias where a score of low, high, or unclear was assigned for each included study. The overall quality of each study was then assessed by grading the 7 bias categories. A score of 3, 1, and 0 were considered as low, unclear, and high risk of bias respectively for each of the seven categories of biases. The scores were averaged for each included study and results are provided in the Table 1. Review authors were not blinded to author and source institution. Any disagreement was resolved by discussion or by third party adjudication.

**Table 1:** Summary characteristics of included studies

Sl No.	Study ID	Country	Intervention- Herbal	Control	Index used*		Average Score of Quality of study
					Plaque	Gingival	
TOOTHPASTE STUDIES							
1	Abhishek 2015	India	*Azadirachta indica*	*non-herbal*	PSL	GLS	16
2	Al-Kholani 2011	Yemen	*camomile*	*conventional*	–	GLS	9
3	Amoain 2010	Iran	*calendula*	*placebo*	PSL	GLS	17
4	Amrutesh 2010	India	*vaikrantha*	*fluoride*	PSL	GLS	17
5	George 2009	India	*camomile*	*fluoride*	TQH	GLS	19
6	Gupta 2012	India	*salvadora persica*	*conventional*	TQH	–	21
7	Habashneh 2017	Jordan	*camomile*	*fluoride*	TQH	GLS	14
8	Mohire 2010	India	*chitosan*	*placebo*	PSL	–	6
9	Olivera 2008	Brazil	*Aloe vera*	*fluoride*	PSL	GLS	19
10	Ozaki 2008	Brazil	*camomile*	*fluoride*	TQH	GLS	21
11	Rao 2008	India	*pumica granatum*	*fluoride*	TQH	GLS	15
12	Tatikonda 2014	India	*azadirachta indica*	*fluoride*	TQH	GLS	16
13	Estafan 1998	USA	*calendula*	*fluoride*	PSL	GLS	10
14	Pereira 2013	Brazil	*lippia sidiodes*	*placebo*	TQH	–	17
15	Pradeep 2012	India	*aloe vera*	*placebo*	TQH	GLS	18
MOUTHRINSE STUDIES							
16	Charles 2004	USA	*essential oils*	*chlorhexidine*	TQH	GLS	16
17	Jain 2017	India	*licorice*	*chlorhexidine*	TQH	GLS	9
18	Lauten 2005	USA	*maleluca*	*chlorhexidine*	PSL	GLS	13
19	Pourabbas 2005	Iran	*camomile*	*chlorhexidine*	TQH	GLS	15
20	Ratika 2014	India	*azadirachta indica*	*chlorhexidine*	PSL	GLS	16
21	Ratika [2] 2014	India	*mango*	*chlorhexidine*	PSL	GLS	16
22	Shetty 2013	India	*azadirachta indica*	*chlorhexidine*	TQH	GLS	19
23	Vangipuram 2016	India	*aloe vera*	*chlorhexidine*	PSL	GLS	21
24	Weijden 1998	Netherlands	*juniper*	*placebo*	PSL	GLS	19

* PSL = Silness and Loe plaque index TQH = Turesky-Gilmore modification of Quigley Hein plaque index GLS = Loe and Silness gingival index

*# Quality of score assessment: No risk – 3, Unclear risk – 1, High risk – 0 (sum of each of the seven biases were taken)*

### Synthesis of results

We performed an evaluation of the heterogeneity of the data using Cochran’s Q statistic, a chi-square test, a threshold *p*-value of less than 0.10 [19]. The consistency of the results was assessed visually using forest plots and by the I^2^ statistic [20]. The I^2^ statistic describes the proportion of variation in point estimates attributable to heterogeneity as compared to sampling error. Subgroup analyses were performed to assess the impact of the HTP on duration of intervention (4 vs. 12 weeks). Forest plots were used for graphic presentation. A ‘Summary of Findings’ Table 2 used the GRADE Profiler software (version 3.6) for the primary outcomes [21].

**Table 2:** Summary of Findings

Outcomes	Intervention (Mean ± SD)	Control (Mean ± SD)	No of Participants (studies)	Pooled Estimate	Ref	Quality of the evidence (GRADE)
HTP Dental Plaque. Follow-up: Short term	The mean short-term effects HTP in the intervention groups was 0.52 ± 0.33	The mean short-term effects of NHTP in the control groups was 0.31 ± 0.21	712 (11 studies)	1.95 higher (0.97, 2.93)	Fig. 2 A	⊕ ⊕ ⊝⊝ low
HTP Dental Plaque. Follow-up: Long term	The mean long-term effects of HTP in the intervention groups was 1.02 ± 0.68	The mean long-term effects of NHTP in the control groups was 0.80 ± 0.50	166 (4 studies)	0.89 higher (−0.93, 2.72)	Fig. 2 A	⊕ ⊕ ⊕⊝ moderate
HTP Gingival inflammation. Follow-up: Short term	The mean short-term effects of HTP in the intervention groups was 0.41 ± 0.30	The mean short-term effects of NHTP in the control groups was 0.32 ± 0.19	410 (10 studies)	0.09 higher (− 0.14, 0.00)	Fig. 2 B	⊕ ⊕ ⊝⊝ low
HTP Gingival Inflammation. Follow-up: Long term	The mean long-term effects of HTP in the intervention groups was 0.50 ± 0.50	The mean long-term effects of NHTP in the control groups was 0.43 ± 0.24	146 (3 studies)	0.07 higher (− 0.23, 0.36)	Fig. 2 B	⊕ ⊕ ⊝⊝ low
HMR Dental Plaque. Follow-up: Short term	The mean short-term effects of HMR in the intervention groups was 0.79 ± 0.49	The mean short-term effects in of NHTP in the control groups was 0.91 ± 0.87	582 (6 studies)	2.93 lower (−6.43, 0.58)	Fig. 3 A	⊕ ⊕ ⊕⊝ moderate
HMR Dental Plaque Follow-up: Long term	The mean long terms effects of HMR in the intervention groups was 0.23 ± 0.51	The mean long terms effects of NHTP in the control groups was 0.33 ± 0.49	285 (5 studies)	2.61 lower (−4.42, −0.80)	Fig. 3 A	⊕ ⊕ ⊕⊝ moderate
HMR Gingival Inflammation. Follow-up: Short term	The mean short-term effects of HMR in the intervention groups was 0.82 ± 0.34	The mean short-term effects of NHTP in the control groups was 0.97 ± 0.54	582 (6 studies)	0.15 lower (−0.32, 0.01)	Fig. 3 B	⊕ ⊕ ⊝⊝ low
HMR Gingival inflammation. Follow-up: Long term	The mean long-term effects of HMR in the intervention groups was 0.22 ± 0.36	The mean long-term effects of NHTP in the control groups was 0.31 ± 0.49	255 (5 studies)	0.09 lower (−0.25, 0.08)	Fig. 3 B	⊕ ⊕ ⊝⊝ low

*HTP herbal toothpaste, NHTP non herbal toothpaste, HMR herbal mouthrinse, NHMR non herbal mouthrinse, Short-term effect 4 weeks, Long Term effects 12 Weeks*

## Results

### Study selection

**Fig. 1:**
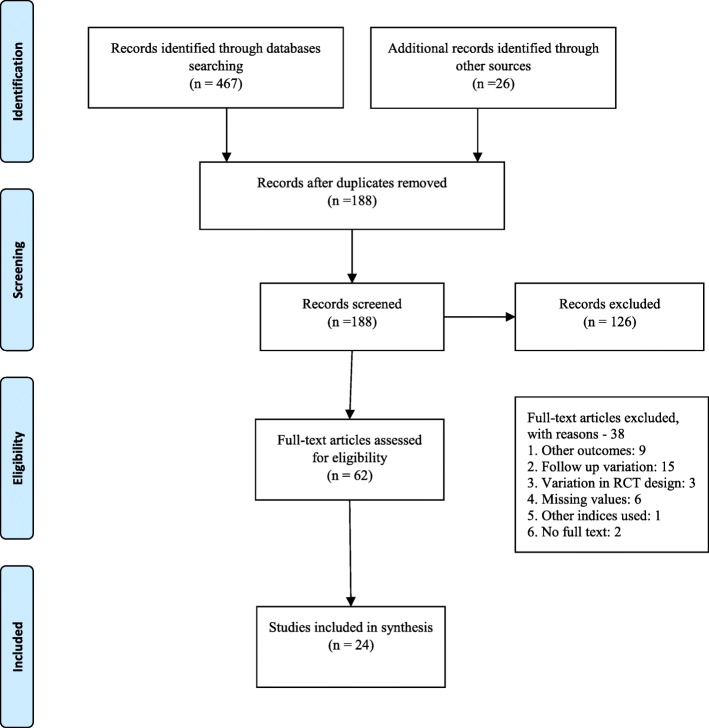
Search strategy

Electronic searches from all sources retrieved 493 citations (Fig. 1). Using titles and abstracts to screen content, 305 citations were excluded duplications. 126 articles were excluded due to non-clinical studies in humans or were reviews or opinion papers. Out of 62 clinical trials, 38 did not meet the inclusion criteria [measured other outcomes [9], follow up period variation [16], variation in RCT design [3], missing values of outcome [6], variation in index used for plaque assessment [1] and full text was not available for two articles] (See Additional file 1: Table S2 for list of excluded trials and reasons). Most studies originated from Southeast Asia and all were in the English language.

### Study description

The 24 RCTs comprising 1597 adults (899 HTP participants and 698 HMR participants) for inclusion in the summary analyses. Selected characteristics of the included studies are shown in Table 1. There were 15 HTP and 9 HMR trials using non-herbal toothpaste (NHTP) or non-herbal mouth rinse (NHMR) as the control arm. Eleven HTP studies [9, 22–31] assessed short-term effects (4-weeks follow up) on dental plaque reduction whereas four studies [31–34] assessed for long-term effects (12-weeks follow up). Ten HTP studies [7, 9, 22–27, 30, 31] assessed short-term effects (4 weeks follow up) on gingival inflammation reduction whereas three studies [31–33] assessed long-term effects. Among the HTP studies, seven and eight studies assessed short-term effects on dental plaque reduction and gingival inflammation reduction, respectively, with fluoridated toothpaste as the control. Six HMR trials each assessed short-term [35–39] and long-term effects [36, 37, 40–42] on dental plaque reduction. Six studies assessed short-term effects on gingival inflammation [35–39] reduction whereas five studies assessed for long-term effects [36, 37, 41, 42].

There was clinical heterogeneity in the herbal ingredients present in toothpastes studied. Four studies [7, 22, 25, 31] assessed *chamomile (Matricaria recutita)*, two studies evaluated *neem* (*Azadirachta indica)* [9, 30], *Aloe vera (Aloe barbadensis)* [23, 33] and *calendula (Calendula officinalis)* [26, 32] *respectively*. Individual studies for *salvoadoral persica* [29], *chitosan* [28], *ajamoda satva (Apium graveolens*) [24], *lippia sidiodes (Pepper-rosmarin)* [34] and *vaikrantha bhasma (Dolichos biflorus) were also conducted* [27]. Eight HTP studies [22–25, 27, 30–32] used fluoride as the control, whereas four studies [26, 28, 33, 34] used placebo with the rest using non-herbal, non-fluoride OTC toothpastes. Six studies [9, 23, 26–28, 32] assessed dental plaque using the Silness and Löe Plaque Index [43] whereas eight studies [22, 24, 25, 29–31, 33, 34] assessed dental plaque using the Turesky-Gilmore modification of the Quigley Hein Plaque [44] Index in HTP studies. All studies assessed gingival inflammation by Silness and Löe Gingival Index [45].

Every HMR study had a different herbal ingredient, with the exception of two trials which had Neem (*Azadirachta indica)* as the active ingredient [36, 37]. Eight of these had chlorhexidine as the control while one had placebo. Five studies assessed dental plaque using the Silness and Löe Plaque Index whereas four studies assessed dental plaque using the Turesky-Gilmore modification of Quigley Hein Plaque Index in HMR studies. All studies assessed gingival inflammation by Silness and Löe Gingival Index. Clinical outcomes in all studies were measured as continuous variables reported as mean ± SD.

### Risk of Bias assessments

A synthesis of the assessment of the methodological quality items (authors’ judgement of risk of bias for each included study) is presented in Additional file 1: Figure. S1. Three studies showed low risk of bias [22, 29, 38], seven studies had unclear risk [23, 25–27, 34, 36, 40] and the remainder were high risk. Additional file 1: Figure. S2 depicts a risk of bias graph, illustrating the authors’ judgements about each risk of bias item presented as percentages across all included studies. Among all, allocation concealment or selection bias and blinding of the participants had higher proportions of bias across the studies.

#### Synthesis of results - effect of interventions

### Herbal toothpaste

**Fig. 2:**
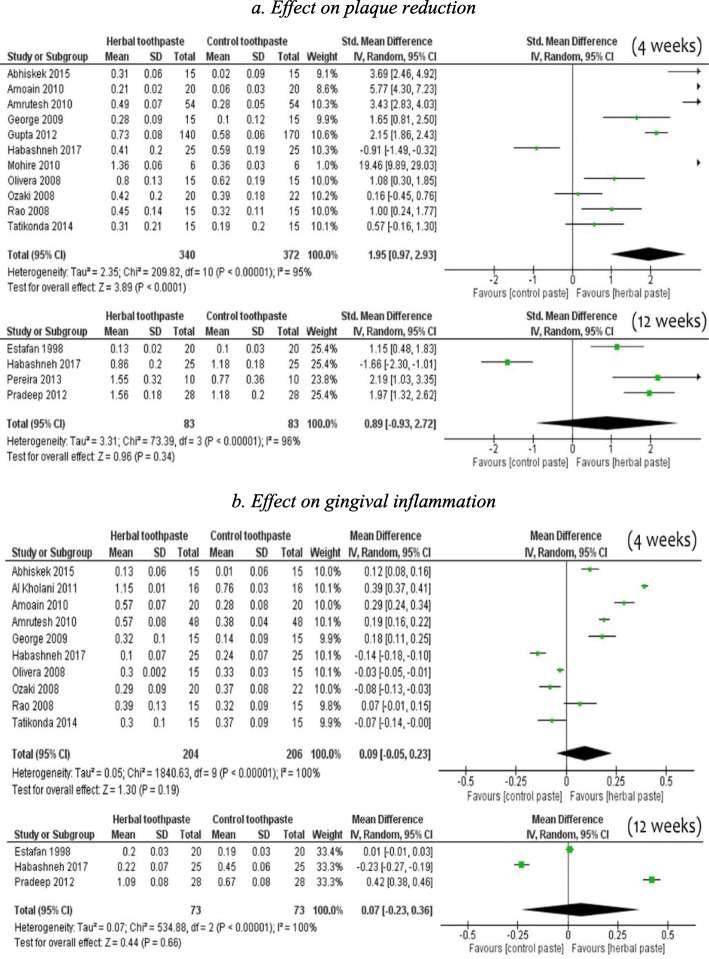
Comparison of herbal toothpaste with non-herbal toothpaste (all controls) 2**a**. Effect on plaque reduction 2**b**. Effect on gingival inflammation

Overall, in 11 pooled studies involving 712 adults (Table 2), participants using HTP were more likely to experience a reduction in dental plaque scores during a four-week period compared to those using NHTP [SMD 1.95, 95% CI (0.97 to 2.93)], but there was substantial heterogeneity (95%) across studies (Fig. 2-2a). However, 4 trials studying long-term effects did not favour HTP for reduction in dental plaque [SMD 0.89, 95% CI (− 0.93 to 2.72)]. Regarding gingival inflammation, for both short-term [SMD 0.09, 95% CI (− 0.14 to 0.00), 10 studies] and long-term effects [SMD 0.07, 95% CI (− 0.23 to 0.36), 3 studies], the pooled results did not significantly favour HTP when compared to NHTP (Fig. 2-2b).

The significant difference in Plaque Index reduction that was found at 4 weeks between HTP and NHTP was investigated with a sub group analysis. The controls (NHTP) were divided into non-fluoride toothpaste and fluoridated toothpaste.

**Fig. 3:**
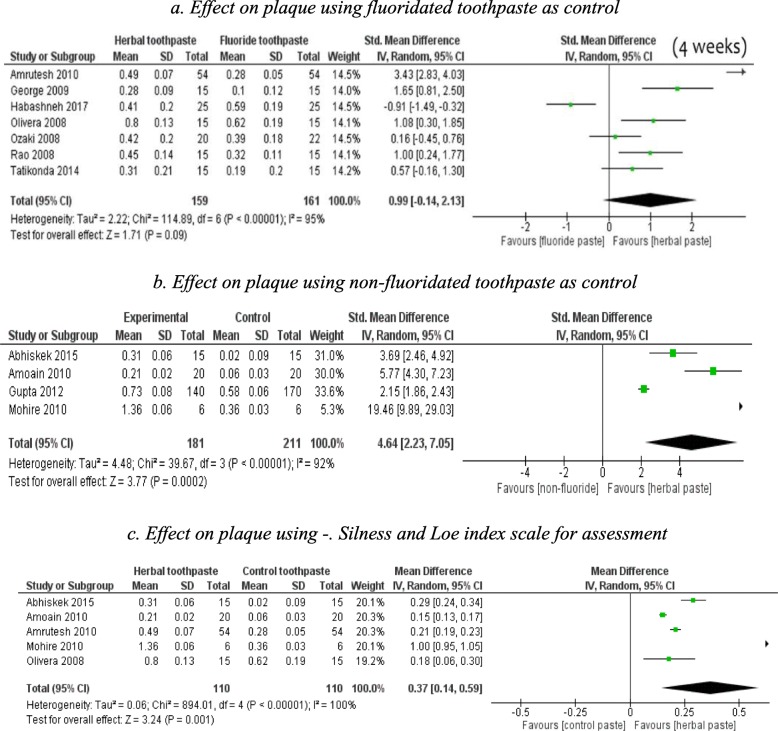
Subgroup analysis; comparison of herbal toothpaste with non-herbal toothpaste at 4 weeks follow-up 3**a**. Effect on plaque using fluoridated toothpaste as control 3**b**. Effect on plaque using non-fluoridated toothpaste as control 3**c**. Effect on plaque using -. Silness and Loe index scale for assessment

HTP was not superior over fluoride toothpaste (SMD 0.99, 95% CI − 0.14 to 2.13, 7 studies, short-term) in reducing dental plaque at 4 weeks (Fig. 3-3a). however, it was favoured to reduce dental plaque over non-fluoride toothpaste (SMD 4.64., 95% CI (2.23, 7.05), 4studies] (Fig. 3-3b). In another subgroup analysis, HTP was favoured over NHTP when short-term studies used the Silness and Löe Index [MD 0.37, 95% CI (0.14 to 0.59), 5 studies] in reducing dental plaque (Fig. 3-3c). There was significantly greater reduction in Plaque was observed for HTP compared to non-fluoride toothpastes, but not with fluoride toothpastes;

### Herbal mouth rinse

**Fig. 4:**
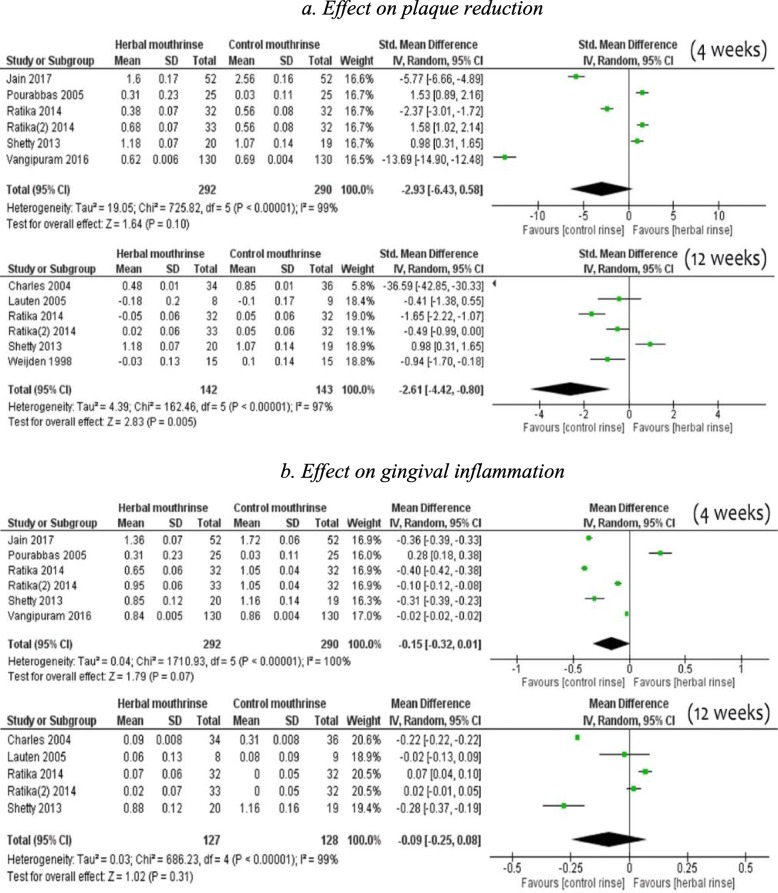
Comparison of herbal Mouth rinse vs non-herbal Mouth rinse 4**a**. Effect on plaque reduction 4**b***.* Effect on gingival inflammation

There was no difference in mean reduction of dental plaque [SMD -2.93, 95% CI (− 6.43 to 0.58), 6 studies, 582 participants] by HMR compared to NHMR for short-term use (Fig. 4-4a). However, there was substantial evidence of mean reduction of dental plaque by users of NHMR compared to HMR in 6 studies [SMD -2.61, 95% CI (− 4.42 to − 0.80), 285 participants) at 12 weeks. Regarding gingival inflammation, for both short-term [SMD -0.15, 95% CI (− 0.32 to 0.01), 6 studies] and long-term effects [SMD -0.09, 95% CI (− 0.25 to 0.08), 6 studies], the pooled findings did not significantly favour NHMR when compared to HMR (Fig. 4-4b).

## Discussion

Despite the fact that most individuals claim to brush their teeth at least twice a day, the prevalence of gingivitis and chronic periodontitis remains high in most populations [46]. The maintenance of an effective level of plaque control is clearly difficult using conventional mechanical procedures and dentifrices and yet, from a therapeutic perspective, it is currently the only realistic means of improving the periodontal health of populations. We assessed whether herbal toothpastes improved the effectiveness of plaque control and gingival health in comparison to non-herbal toothpaste. Overall, our findings suggest that HTP is superior to NHTP at removing supra-gingival plaque at short term use of 4 weeks, but there is no difference at long-term use of 12 weeks.

In this review, HTP contained a variety of herbs or plant extracts, which accounts for substantial clinical heterogeneity. The term ‘herbal’ is used to refer collectively to all ingredients that are botanicals or extracts and these ingredients should not be inferred as necessarily therapeutic within the composition of the product [47]. For example, if an herb like *Aloe vera* or *neem* is added to a toothpaste, the component of *neem* or *Aloe vera* which might act against the cariogenic microflora is unknown or has been isolated. Hence, it can be argued that there is a plurality of effect in herbal or botanical extracts making its action non-specific. Studies have shown that herbal extracts are indicated for their cleansing, astringent, anti-microbial, and refreshing properties which are non-specific actions in body [31, 48–51]. However, the anti-plaque efficacy or reduction of gingival inflammation by fluoride is specific to dental plaque and oral microorganisms. Therefore, it could be concluded that the herbal toothpaste may not exert significant therapeutic effects on plaque and gingivitis beyond that of a conventional commercial dentifrice.

HTP was effective in reducing dental plaque in studies having non-fluoridated toothpaste as the control, and the effect was statistically significant. Dental plaque is a significant risk factor for the development of dental caries and periodontal disease [52]. One proposed mechanism of action regarding the active ingredients of herbal dentifrices is penetration of the biofilm and prevention of plaque accumulation, thereby potentially preventing the colonization of oral bacteria on the tooth surfaces [53]. However, very few studies have evaluated the microbial efficacy of commercially available herbal dentifrices against oral microflora [26, 49]. Studies using the Silness and Löe Index for measuring dental plaque showed statistically significant plaque reduction at 4 weeks, but not at 12 weeks duration. Ideally long-term action is an important indicator of potency of the toothpaste, however, in this finding suggest, there may be methodological bias related outcome assessment measures and plurality of herbal ingredients.

There was little difference in the use of HMR compared to NHMR for reduction in dental plaque or gingival inflammation regardless of study duration. In all trials, NHMR were based on chlorhexidine, which has been proven to be a specific agent against oral microorganisms associated with dental caries and periodontal disease. There is strong evidence for the anti-plaque and anti-gingivitis effects of chlorhexidine mouth-rinse used as an adjunct to regular oral hygiene in patients with periodontal disease [54]. In this systematic review, patients using chlorhexidine experienced a 33% reduction in plaque and a 26% in gingivitis. Chlorhexidine is effective against an array of microorganisms including gram-positive and gram-negative bacteria, fungi, yeast and viruses. It is bacteriostatic at low concentration and bactericidal at high concentrations [55]. In a meta-analysis comparing the effect of essential oil mouth-rinses with chlorhexidine, it was found that chlorhexidine was superior to essential oils in terms plaque reduction while there were no significant differences in gingivitis reduction [56].

The need for an oral rinse to be retained in the oral cavity to maintain potency over an extended length of time has been debated. An antimicrobial agent needs sufficient substantivity (defined as the persistence of the effect of its active ingredient) to inhibit or kill a microorganism [57]. Chlorhexidine, with a substantivity of 12 h, is highly effective, whereas the substantivity of herbal mouth rinses is unknown. Based on the results of this review, there is not enough statistically significant evidence to suggest that herbal oral rinses had a greater effect in reducing gingival index scores or plaque scores. Mouth rinses are generally prescribed for two different conditions; maintenance of oral health in patients with good oral hygiene and to recover from local (gingivitis, periodontitis, surgical treatments, radiotherapy) and systemic (alteration of the immune response, chemotherapy) disorders. Our findings do not support a recommendation for the use of herbal mouth rinses for daily use or for any specific condition, unlike chlorhexidine mouth rinse which is well supported by research. However, taking into consideration the long-term adverse effects with the use of chlorhexidine, conditionally HMR can be recommend as an alternative.

Herbal medicines are plant-derived materials or products with therapeutic properties used in folk medicine, involving both Eastern and Western medical traditions. The use of these products in the prevention and treatment of oral conditions has increased recently and could benefit low socio-economic rural communities especially in low income countries. Herbal extracts have received special attention because they are non-synthetic or “organic” in nature. Consumers who use herbal products often view these products as being safer than products that have “chemicals” although there are reports of allergy/hypersensitivity reactions resulting from herbal and conventional toothpastes. The wide variety of formulations hampers the ability to identify whether a clinical outcome is related to the herbal or other active agents. Research on the side effects of these formulations is still lacking. Nevertheless, due to the demand for natural products, there is a thriving market for herbal oral care products.

Herbal toothpastes or mouth rinses should be tested for equivalence in efficacy against the use of a positive control using standard products containing fluoride or chlorhexidine rather than the use of non-fluoride toothpastes or non-chlorhexidine mouth rinses. Future research would benefit from a uniform method of assessment for clinical effectiveness of plaque and gingivitis using these products. Currently, heterogeneity in methodology and evaluation, including the duration of follow-up and assessment is hindering the development of synthesized evidence to determine product effectiveness. Finally, there is a lack of a uniform reporting, including any adverse events associated with the use of experimental herbal products, although reporting standards for RCTs exist (e.g., CONSORT). Reporting standards are very important for clinical information systems where evidence translation solely depends on RCTs. However, RCTs testing the herbal products evaluations poses complex problem for clinical or health information due to variation in comparison of two different system of medicine like western with alternative medicine. So, there is a need for reporting guidelines for herbal molecules product studies, which would enhance the knowledge transformation of the research evidence into policy.

## Conclusion

Herbal toothpaste appears to be equally effective as non-herbal toothpaste, but not superior to fluoride toothpaste. The herbal mouth rinses were found not to be superior to chlorhexidine mouth rinses. The quality of evidence appears to be low/very low to recommend them as a substitute to more conventional OTC oral hygiene products.

## Supplementary information

**Additional file 1 Figure S1** Review authors' judgements about each risk of bias item for each included study. **Figure S2** Risk of bias graph: review authors' judgements about each risk of bias item presented as percentages across included studies. **Table S1 Table S2**. List of excluded studies and reasons for exclusion

## Data Availability

” Not applicable”.

## Abbreviations

BD: Bruce Dye
CJ: Chandrashekar Janakiram
HMR: Herbal mouth rinse
HTP: Herbal toothpaste
MD: Mean Deviation
NHMR: Non-herbal mouth rinse
NHTP: Non-herbal toothpaste
OTC: Over the Counter
PF: Paul Fontelo
RV: Ramanarayanan Venkitachalam
SMD: Standard Mean Deviations
TI: Timothy Iafolla
