# Supplementary material for: Effectiveness of herbal oral care products in reducing dental plaque & gingivitis – a systematic review and meta-analysis
Source: BMC Complement Med Ther. 2020 Feb 11;20:43. doi: 10.1186/s12906-020-2812-1 (PMC7076867; doi:10.1186/s12906-020-2812-1)
Supplement: Supplementary file 1 — Additional file 1 Figure S1 Review authors' judgements about each risk of bias item for each included study. Figure S2 Risk of bias graph: review authors' judgements about each risk of bias item presented as percentages across included studies. Table S1 Table S2. List of excluded studies and reasons for exclusion [file 12906_2020_2812_MOESM1_ESM.docx]

Supplemental Tables/Figures


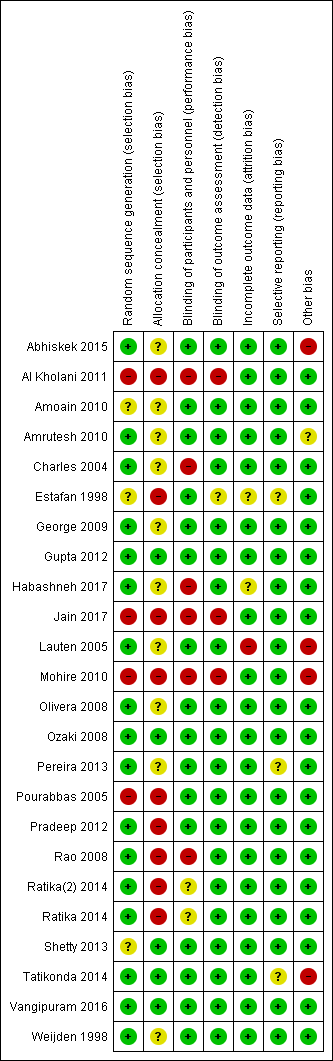


Supplemental Figure 1: review authors' judgements about each risk of bias item for each included study.


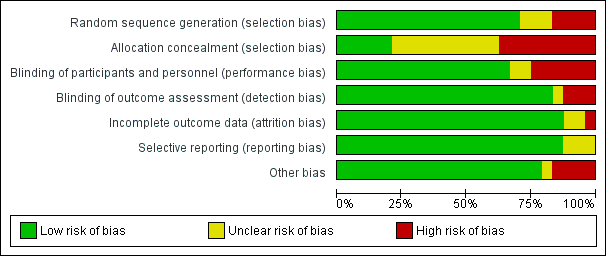


Supplemental Figure 2: Risk of bias graph: review authors' judgements about each risk of bias item presented as percentages across included studies.

Supplemental Table 1 :Search strategy

Search ((herb OR herbal OR herbs OR “all natural” OR natural OR organic) AND ("Mouthwashes"[Mesh:NoExp] OR “mouthwash”[tiab] OR “mouthwashes”[tiab] OR "mouthwashes"[Pharmacological Action] OR “oral rinse”[tiab] OR “mouth rinse”[tiab] OR "Toothpastes"[Mesh] OR toothpaste[tiab] OR toothpastes[tiab] OR “tooth paste”[tiab] OR “tooth pastes”[tiab] OR “tooth powder”[tiab] OR “tooth powders”[tiab] OR “teeth powders”[tiab] OR “teeth powder”[tiab] OR “oral care product”[tiab] OR “oral care products”[tiab]) AND (Plaque[tiab] OR "dental plaque"[tiab] OR gingivitis[tiab] OR “Dental caries”[mesh] OR “dental caries”[tiab] OR "Gingivitis"[Mesh] OR "Dental Plaque"[Mesh]))

Supplemental Table 2. List of excluded studies and reasons for exclusion

| Sl No. | Studies | Reason for exclusion |
| --- | --- | --- |
| 1 | Aftab Alam, Nayyar Parvez, Suman Yadav, Raman Dang, JP Shethy, 2008. Comparative Evaluation of Herbal mouthrinse, herbal toothpaste gel and chlorhexidine mouthrinse for cariogenic bacteria. Cont. J Pharmacol. Toxicol. Res. 2, 19–26. | Microbial study |
| 2 | Aravinth, V., Narayanan, M.A., Kumar, S.R., Selvamary, A.L., Sujatha, A., 2017. Comparative evaluation of salt water rinse with chlorhexidine against oral microbes: A school-based randomized controlled trial. J. Indian Soc. Pedod. Prev. Dent. 35, 319. | 5 days follow up |
| 3 | Arjun TN, Abhishek Gouraha, Ashish Maheshwari, Kalpesh Chavan, 2015. Efficacy of herbal dentifrice in the reduction of dental caries compared against commercially available fluoride containing dentifrice: An experimental trial. World J. Pharm. Pharm. Sci. 4, 800–807. | Microbial study |
| 4 | Azaripour, A., Mahmoodi, B., Habibi, E., Willershausen, I., Schmidtmann, I., Willershausen, B., 2017. Effectiveness of a miswak extract‐containing toothpaste on gingival inflammation: a randomized clinical trial. Int. J. Dent. Hyg. 15, 195–202. | 3 weeks follow up, API and SBI used |
| 5 | Baradari, A.G., Khezri, H.D., Arabi, S., 2012. Comparison of antibacterial effects of oral rinses chlorhexidine and herbal mouth wash in patients admitted to intensive care unit. Bratisl. Lek. Listy 113, 556–560. | Microbial study |
| 6 | Bhat, N., Bapat, S., Asawa, K., Tak, M., Chaturvedi, P., Gupta, V.V., George, P.P., 2015. The antiplaque efficacy of propolis-based herbal toothpaste: A crossover clinical study. J. Nat. Sci. Biol. Med. 6, 364–368. | 1 day follow up |
| 7 | Botelho, M.A., Santos, R.A. dos, Martins, J.G., Carvalho, C.O., Paz, M.C., Azenha, C., Ruela, R.S., Queiroz, D.B., Ruela, W.S., Marinho, G., Ruela, F.I., 2009. Comparative effect of an essential oil mouthrinse on plaque, gingivitis and salivary Streptococcus mutans levels: a double blind randomized study. Phytother. Res. 23, 1214–1219. | 1 week follow up |
| 8 | Chandrahas, B., Jayakumar, A., Naveen, A., Butchibabu, K., Reddy, P.K., Muralikrishna, T., 2012. A randomized, double-blind clinical study to assess the antiplaque and antigingivitis efficacy of Aloe vera mouth rinse. J. Indian Soc. Periodontol. 16, 543–548. | Not full mouth design |
| 9 | Chatterjee, A., Saluja, M., Singh, N., Kandwal, A., 2011. To evaluate the antigingivitis and antipalque effect of an Azadirachta indica (neem) mouthrinse on plaque induced gingivitis: A double-blind, randomized, controlled trial. J. Indian Soc. Periodontol. 15, 398–401. | No values given |
| 10 | Chen, Y., Wong, R.W.K., McGrath, C., Hagg, U., Seneviratne, C.J., 2014. Natural compounds containing mouthrinses in the management of dental plaque and gingivitis: a systematic review. Clin. Oral Investig. 18, 1–16. https://doi.org/10.1007/s00784-013-1033-0 | Systematic review |
| 11 | Chhina, S., Singh, A., Menon, I., Singh, R., Sharma, A., Aggarwal, V. A randomized clinical study for comparative evaluation of Aloe Vera and 0.2% chlorhexidine gluconate mouthwash efficacy on de-novo plaque formation. J. Int. Soc. Prev. Community Dent. 6, 251. | 4 days follow up |
| 12 | Deshpande, R.R., Kachare, P., Sharangpani, G., Varghese, V.K., Bahulkar, S.S., 2014. Comparative evaluation of antimicrobial efficacy of two commercially available dentifrices (fluoridated and herbal) against salivary microflora. Int J Pharm Pharm Sci 6, 72–4. | Microbial study |
| 13 | Dodwad Vidya, Malhotra Sumit, Nayyar Nandini, 2011. Toothpaste wars: To assess the efficacy of a herbal, homeopathic and a conventional toothpaste in the control of plaque and gingivitis - A clinico-biochemical study. Indian J. Stomatol. 2, 91–94. | 3 weeks follow up |
| 14 | Geidel, A., Krüger, M., Schrödl, W., Jentsch, H., 2017. Control of Plaque and Gingivitis by an Herbal Toothpaste - A Randomised Controlled Study. Oral Health Prev. Dent. 15, 407–413. https://doi.org/10.3290/j.ohpd.a38975 | API and OHI given |
| 15 | Goultschin, J., Palmon, S., Shapira, L., Brayer, L., Gedalia, I., 1991. Effect of glycyrrhizin-containing toothpaste on dental plaque reduction and gingival health in humans. A pilot study. J. Clin. Periodontol. 18, 210–212. | No values given ... only graphs |
| 16 | Gupta, N., Sharma, A., Chaudhary, R., 2017. Comparative effect of Herbal and Carbopol formulated dentifrices on established gingivitis. J. Dent. Spec. 5, 31–35. | Std dev not given |
| 17 | Haffajee, A.D., Yaskell, T., Socransky, S.S., 2008. Antimicrobial effectiveness of an herbal mouthrinse compared with an essential oil and a chlorhexidine mouthrinse. J. Am. Dent. Assoc. 1939 139, 606–611. | Microbial study |
| 18 | Hosadurga R, Boloor VA, Rao SN, MeghRani N. 2018. Effectiveness of two different herbal toothpaste formulations in the reduction of plaque and gingival inflammation in patients with established gingivitis - A randomized controlled trial. J. Tradit. Complement. Med. 8:113–119. doi:10.1016/j.jtcme.2017.04.005. | Both arms herbal toothpaste – no controls |
| 19 | Hosamane, M., Acharya, A.B., Vij, C., Trivedi, D., Setty, S.B., Thakur, S.L., 2014. Evaluation of holy basil mouthwash as an adjunctive plaque control agent in a four day plaque regrowth model. J. Clin. Exp. Dent. 6, e491-496. https://doi.org/10.4317/jced.51479 | 4 days follow up |
| 20 | Howshigan, J., Perera, K., Samita, S., Rajapakse, P.S., 2015. The effects of an Ayurvedic medicinal toothpaste on clinical, microbiological and oral hygiene parameters in patients with chronic gingivitis: a double-blind, randomised, placebo-controlled, parallel allocation clinical trial. Ceylon Med. J. 60, 126–132. https://doi.org/10.4038/cmj.v60i4.8219 | Std dev not given. . only graphs |
| 21 | Jayashankar, S., Panagoda, G.J., Amaratunga, E. a. P.D., Perera, K., Rajapakse, P.S., 2011. A randomised double-blind placebo-controlled study on the effects of herbal toothpaste on gingival bleeding, oral hygiene and microbial variables. Ceylon Med. J. 56, 5–9. | Plaque and gingival scores not given . . . only post analysis data |
| 22 | Kamali A, Khazaeli P, 2003. Clinical evaluation of effect of toothpaste , persica, darugarl, on the plaque and gingival index. Majallah--Dandanpizishki 14, 65–72. | No full text |
| 23 | Karim, B., Bhaskar, D.J., Agali, C., Gupta, D., Gupta, R.K., Jain, A., Kanwar, A., 2014. Effect of Aloe vera mouthwash on periodontal health: triple blind randomized control trial. Oral Health Dent. Manag. 13, 14–19. | Std dev values not given |
| 24 | Khairnar, M., Dodamani, A., Karibasappa, G.N., Deshmukh, M., Naik, R., 2016. Comparative Evaluation of Efficacy of Three Different Herbal Toothpastes on Salivary Alkaline Phosphatase and Salivary Acid Phosphatase - A Randomized Controlled Trial. J. Clin. Diagn. Res. JCDR 10, ZC69-ZC73. | ACP, ALP study |
| 25 | Khalessi, A.M., Pack, A.R.C., Thomson, W.M., Tompkins, G.R., 2004. An in vivo study of the plaque control efficacy of Persica: a commercially available herbal mouthwash containing extracts of Salvadora persica. Int. Dent. J. 54, 279–283. | 3 weeks follow up |
| 26 | Kumari, M., Naik, S.B., Martande, S.S., Pradeep, A.R., Singh, P., 2016. Comparative efficacy of a herbal and a non-herbal dentifrice on dentinal hypersensitivity: a randomized, controlled clinical trial. J. Investig. Clin. Dent. 7, 46–52. | Hypersensitivity study |
| 27 | Mitra Deepika, Shah Srishti, Shah Rohit, Rodrigues Silvia, Pathare Pragalbha, Vijayakar Harshad, 2015. Evaluation of the Clinical Efficacy of a Herbal Toothpaste in Comparison with a Triclosan Containing Toothpaste in a Population of Dental College Students – A Double-blind Randomized Controlled Trial. Indian J. Contemp. Dent. 3, 67–70 | 3 days follow up |
| 28 | Namiranian, H., Serino, G., 2012. The effect of a toothpaste containing aloe vera on established gingivitis. Swed. Dent. J. 36, 179–185. | No full text |
| 29 | Pannuti, C.M., Mattos, J.P. de, Ranoya, P.N., Jesus, A.M. de, Lotufo, R.F.M., Romito, G.A., 2003. Clinical effect of a herbal dentifrice on the control of plaque and gingivitis: a double-blind study. Pesqui. Odontol. Bras. Braz. Oral Res. 17, 314–318. | 3 weeks follow up |
| 30 | Parwani, S.R., Parwani, R.N., Chitnis, P.J., Dadlani, H.P., Prasad, S.V.S., 2013. Comparative evaluation of anti-plaque efficacy of herbal and 0.2% chlorhexidine gluconate mouthwash in a 4-day plaque re-growth study. J. Indian Soc. Periodontol. 17, 72–77. | 4 days follow up |
| 31 | Peck MT, Charlene WJ, Lawrence XG, Johan Marnewick, Abdul Majeed, 2011. An in-vitro analysis of the antimicrobial efficacy of herbal toothpastes on selected primay plaque colonizers. Int. J. Clin. Dent. Sci. 2, 28–32. | Invitro study |
| 32 | Radafshar, G., Mahboob, F., Kazemnejad, E., 2010. A study to assess the plaque inhibitory action of herbal-based toothpaste: A double blind controlled clinical trial. J. Med. Plants Res. 4, 1182–1186 | 4 days follow up |
| 33 | Rezaei, S., Rezaei, K., Mahboubi, M., Jarahzadeh, M.H., Momeni, E., Bagherinasab, M., Targhi, M.G., Memarzadeh, M.R., 2016. Comparison the efficacy of herbal mouthwash with chlorhexidine on gingival index of intubated patients in Intensive Care Unit. J. Indian Soc. Periodontol. 20, 404–408. | 4 days follow up |
| 34 | Salgado, A.D.Yunes, Maia, J.L., Pereira, S.L. da S., de Lemos, T.L.G., Mota, O.M. de L., 2006. Antiplaque and antigingivitis effects of a gel containing Punica granatum Linn extract: a double-blind clinical study in humans. J. Appl. Oral Sci. Rev. FOB 14, 162–166. | 3 weeks follow up |
| 35 | Srinivasa, S., Bhojraj, N., Srilatha, K.T., 2011. A comparative evaluation of a commercially available herbal and non-herbal dentifrice on dental plaque and gingivitis in children-A residential school-based oral health programme. J. Dent. Oral Hyg. 3, 109–113. | 3 weeks follow up |
| 36 | Tandon, S., Gupta, K., Rao, S., Malagi, K.J., 2010. Effect of Triphala mouthwash on the caries status. Int. J. Ayurveda Res. 1, 93–99. | Caries study |
| 37 | Jalaluddin M , Rajasekaran UB , Paul S , Dhanya RS , Sudeep CB and Adarsh VJ. Comparative Evaluation of Neem Mouthwash on Plaque and Gingivitis: a Double-blind Crossover Study. Journal of contemporary dental practice, 2017, 18(7), 567-571. | 15 days follow up |
| 38 | K. S. Vinod, K. S. Sunil, Priyanka Sethi, Ram Chand Bandla, Subhasini Singh, and Deepak Patel. A Novel Herbal Formulation versus Chlorhexidine Mouthwash in Efficacy against Oral Microflora. J Int Soc Prev Community Dent. 2018 Mar-Apr; 8(2): 184–190. | 14 days follow up |
